# Supplementary material for: Lots of movement, little progress: a review of reptile home range literature
Source: PeerJ. 2021 Jul 20;9:e11742. doi: 10.7717/peerj.11742 (PMC8300531; doi:10.7717/peerj.11742)
Supplement: Supplemental Information 1 [file peerj-09-11742-s001.docx]

**Table S1. List of other home range estimation methods recorded.**

| **Method used** |
| --- |
| a-LoCoH |
| alpha-hull |
| linear range size |
| linear range span |
| river channel area |
| mid stream linear distance |
| harmonic mean |
| Bivariate Normal |
| kernel density ellipses |
| minimum bounded geometry |
| linear home range |
| other |
| bivariate normal |
| k-LoCoH |
| knn-LoCoH |
| 3D-MCP |
| Two-dimensional footprint area |
| adjusted MCP |
| outlier-exclusion cores;incremental cluster polygons |
| unweighted harmonic surface area |
| Brownian bridge kernel |
| f-LoCoH |
| grid cell counts along linear path |
| Bivariate normal ellipse |
| Mean squared displacement |
| Minimum areal home range |
| minimum linear home range |
| t-LoCoH |
| complex linear home range |
| aquatic MCP |
| incremental cluster polygons |
| concave polygons |
| ellipse |
| clusters |
| temporally corrected harmonic mean |
| dynamic Brownian Brownian Bridge Movement Models, Brownian Brownian Bridge Movement Models |
| centre of mass |
| One-mode Bivariate Normal |
| Two-mode Bivariate Circle |
| Two-mode Bivariate Normal Mix |
| mean squared distance |
| bivariate normal-density kernel |
