# Supplementary figures and images for: Lots of movement, little progress: a review of reptile home range literature
Source: PeerJ. 2021 Jul 20;9:e11742. doi: 10.7717/peerj.11742 (PMC8300531; doi:10.7717/peerj.11742)

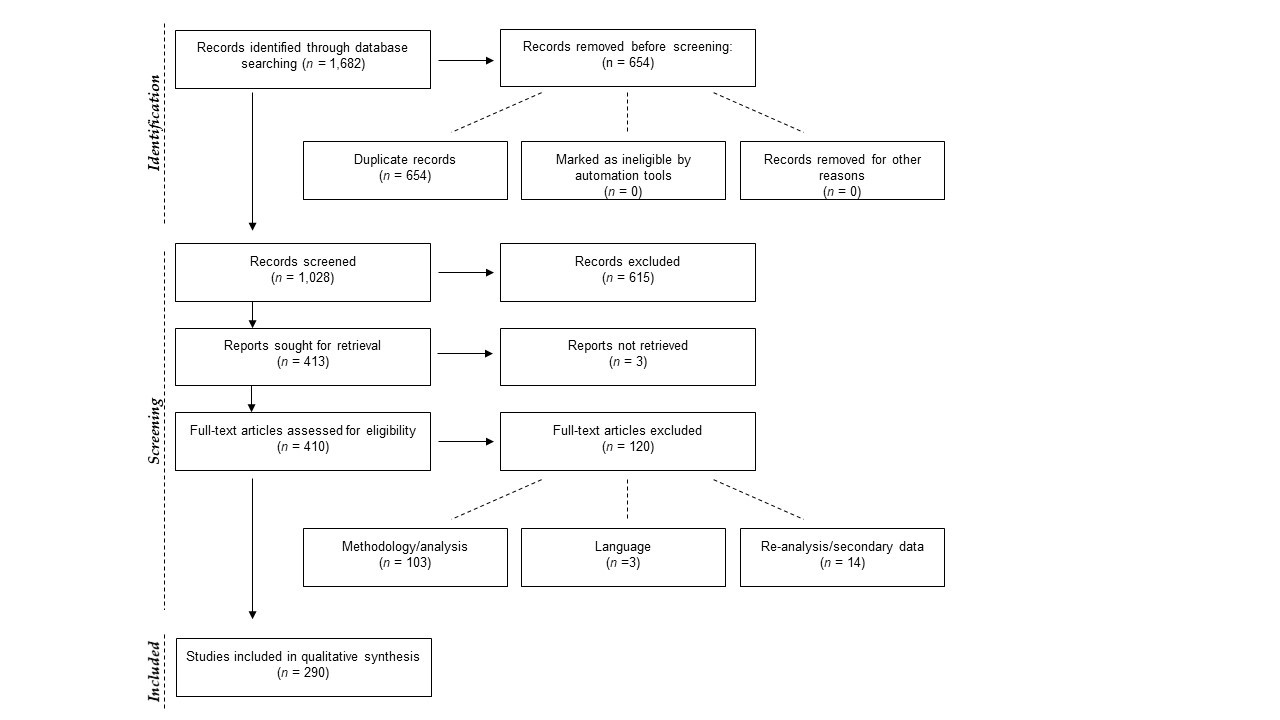

Supplement: Supplemental Information 2 [file peerj-09-11742-s002.jpg]

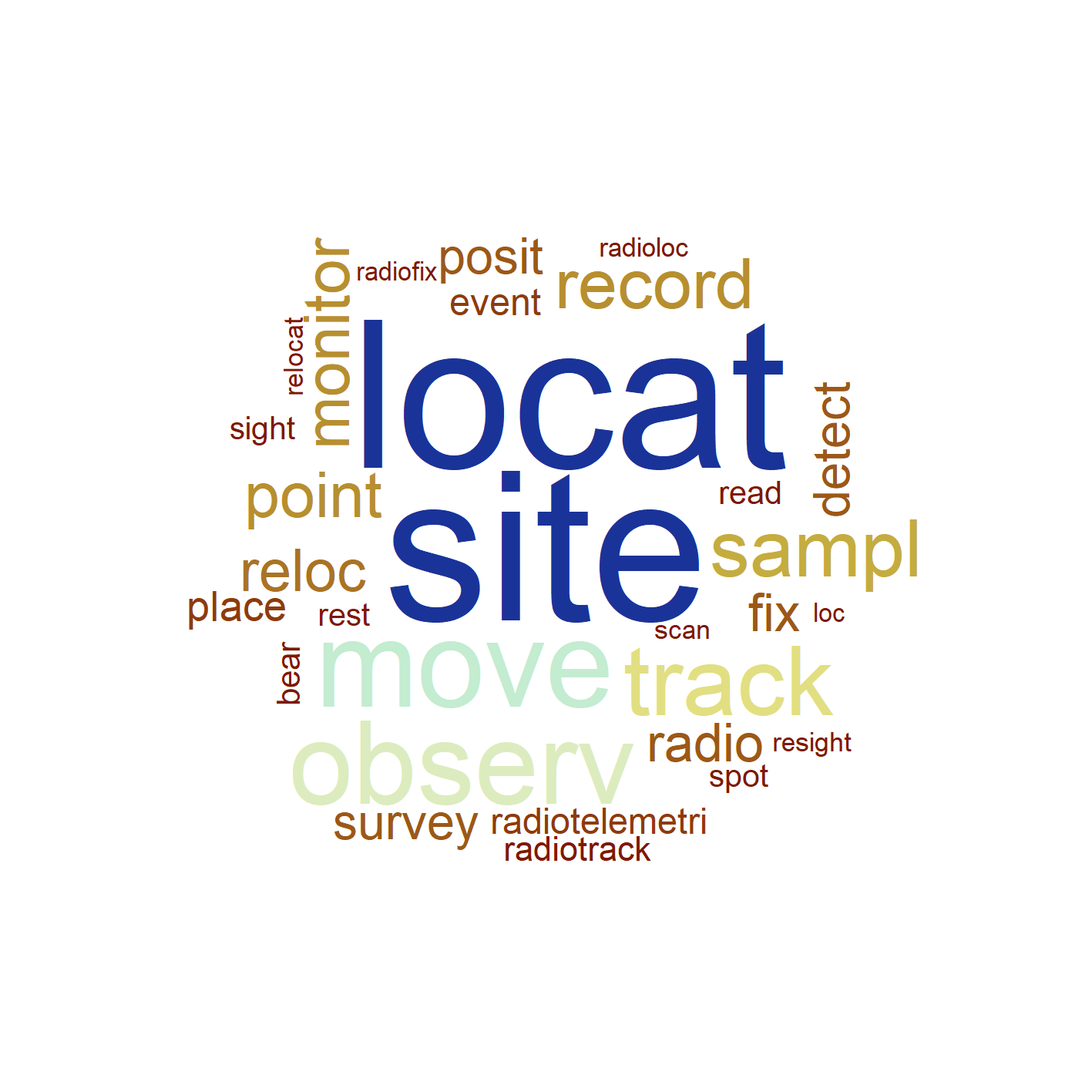

Supplement: Supplemental Information 3 [file peerj-09-11742-s003.png]

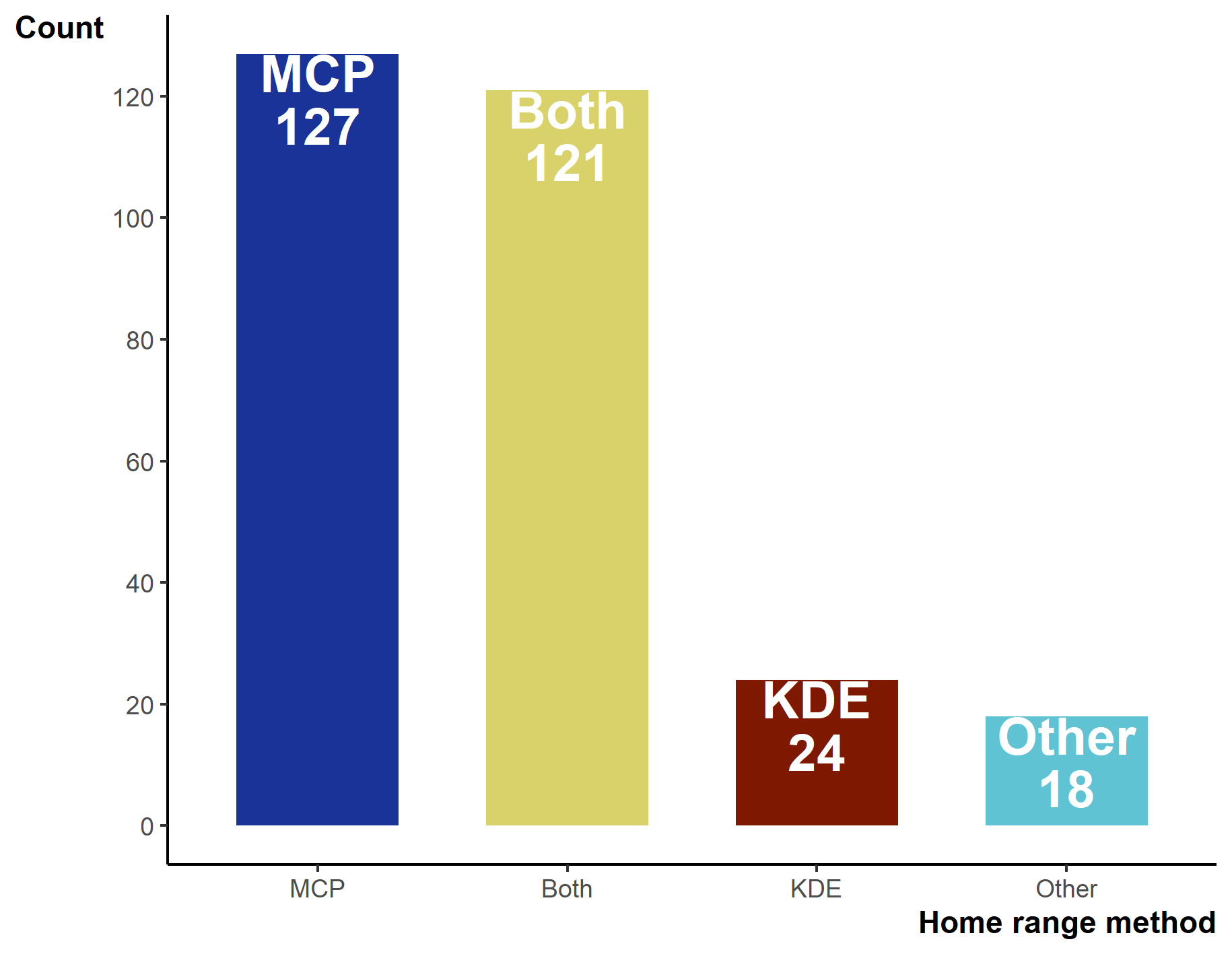

Supplement: Supplemental Information 4 [file peerj-09-11742-s004.png]

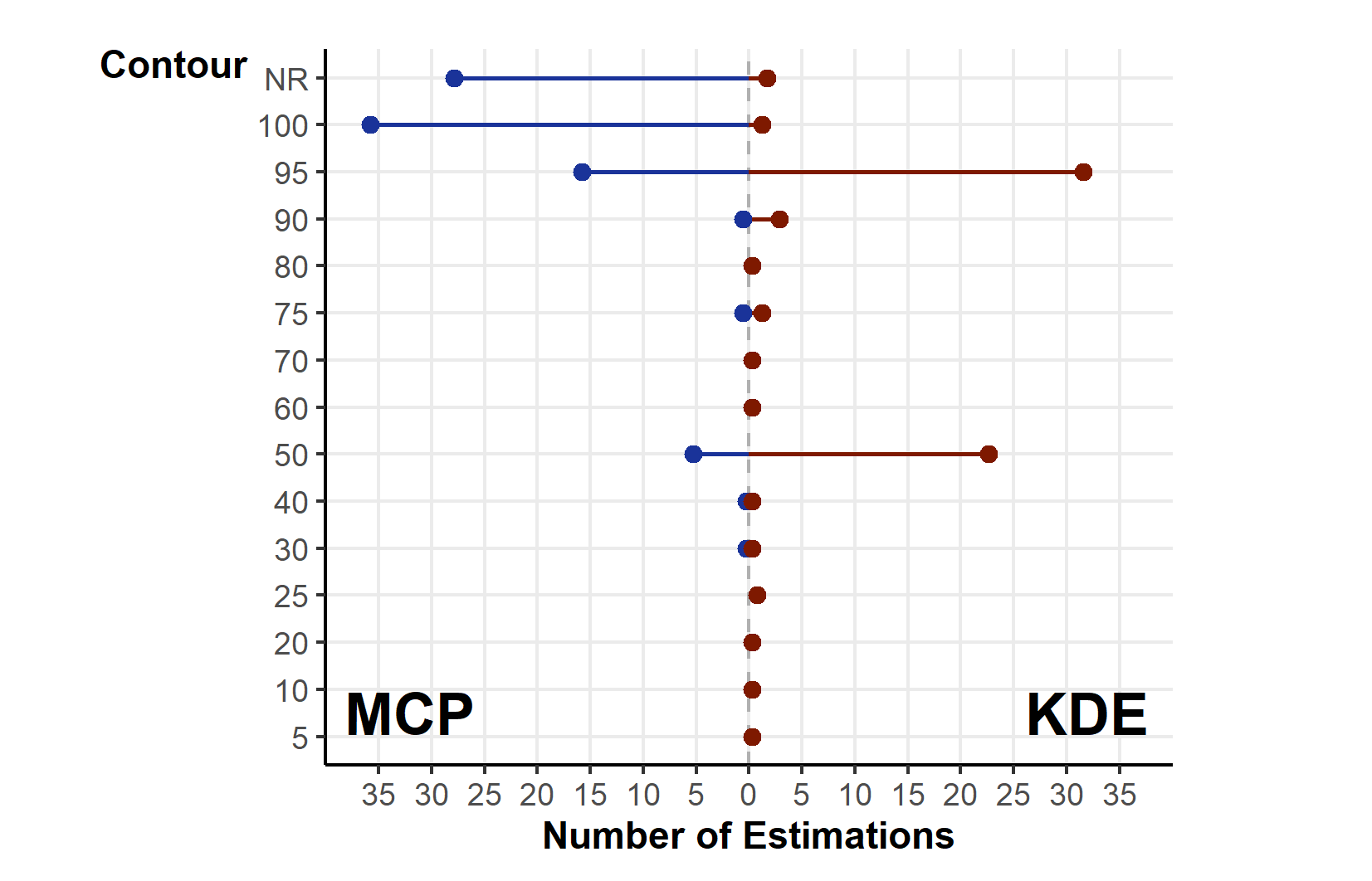

Supplement: Supplemental Information 5 [file peerj-09-11742-s005.png]

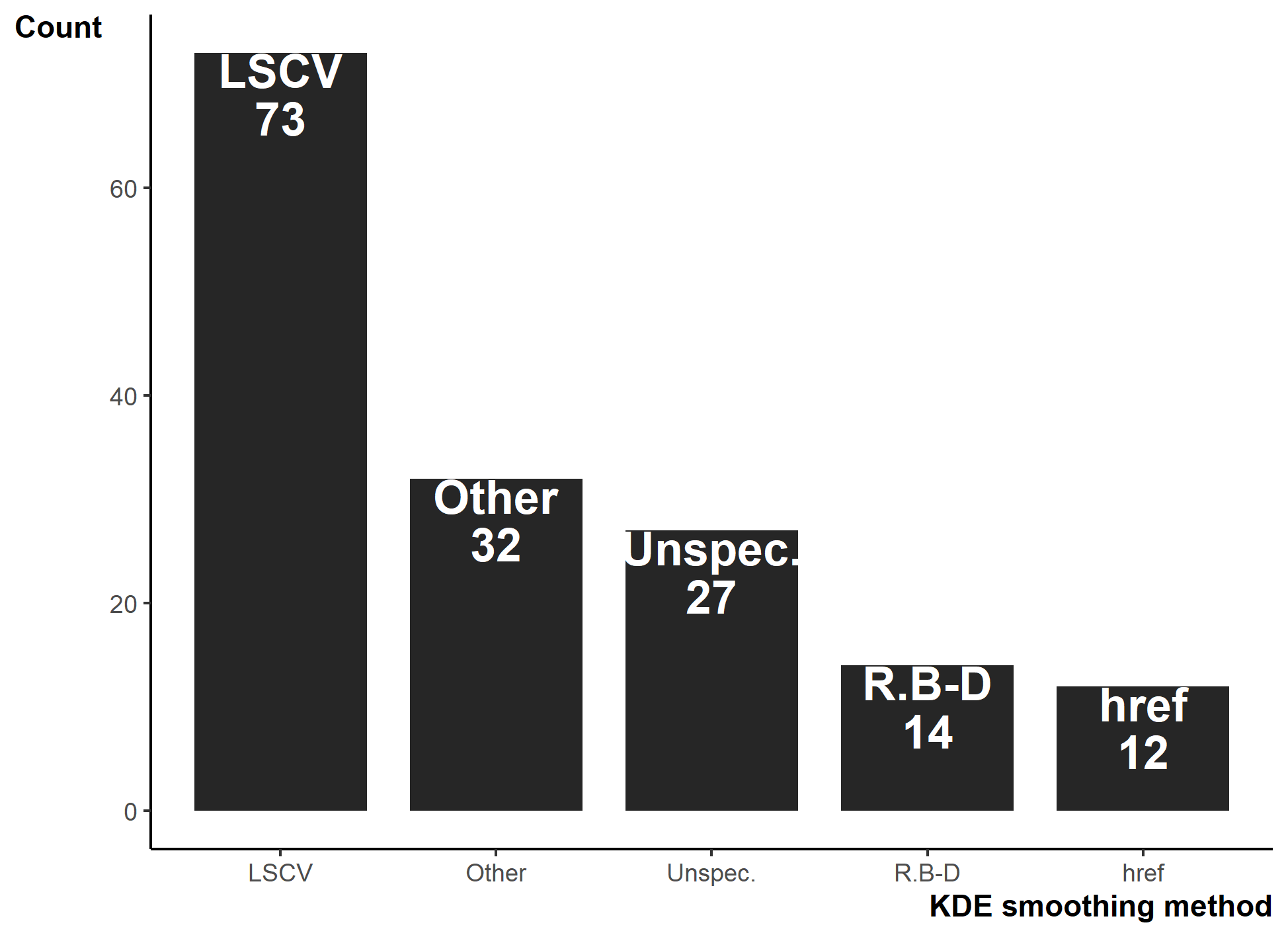

Supplement: Supplemental Information 6 — Unspec. are instances where the smoothing factor (h) was not explicitly stated. R.B-D are cases where studies manipulated h to ensure KDE area output equaled MCP area estimation as suggested by (Row & Blouin-Demers, 2006). [file peerj-09-11742-s006.png]

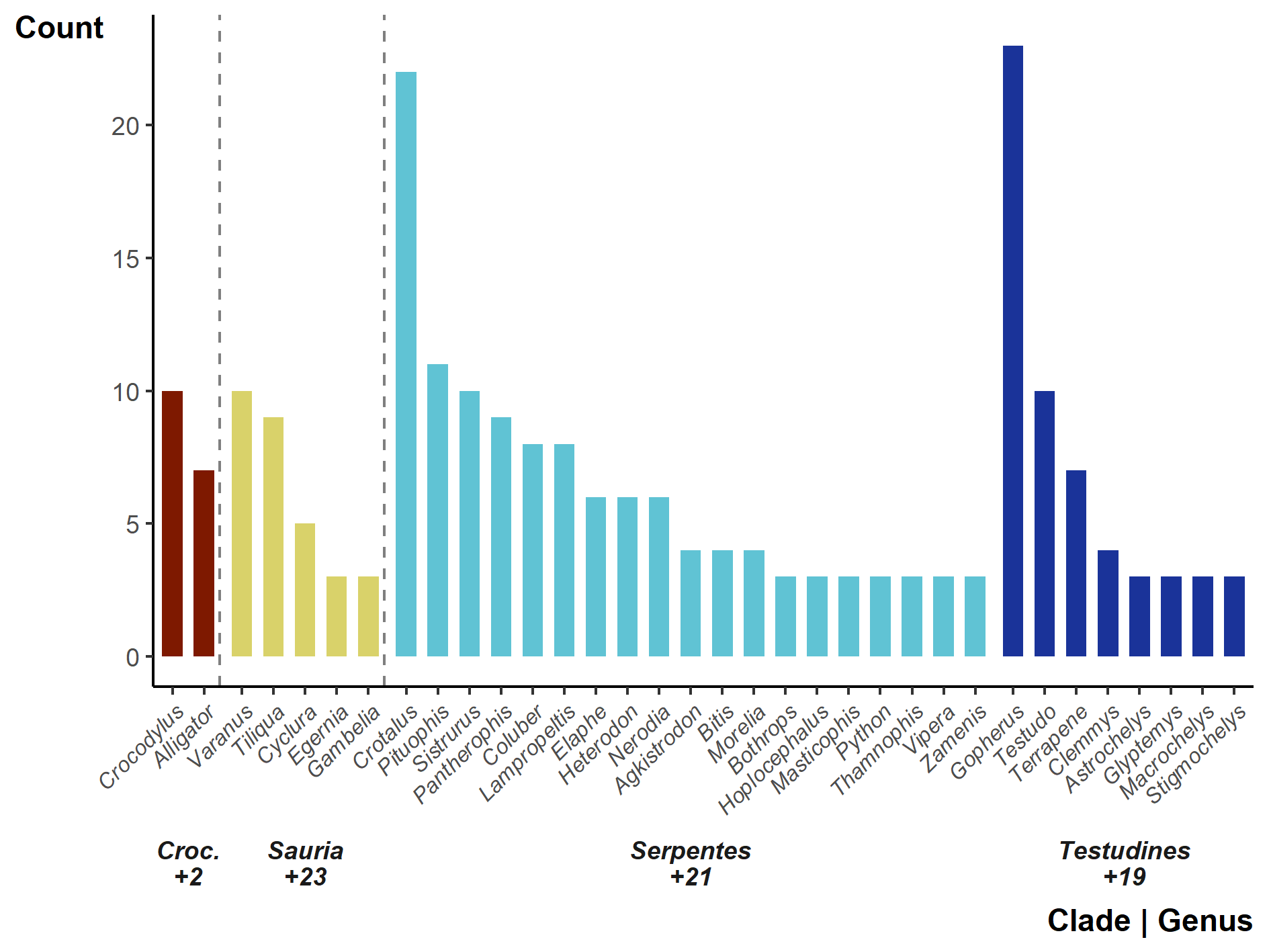

Supplement: Supplemental Information 7 — Numbers below clades indicate the number of genera not listed (i.e., genus only appearing in two or fewer studies). [file peerj-09-11742-s007.png]
