# Supplementary material for: Lots of movement, little progress: a review of reptile home range literature
Source: PeerJ. 2021 Jul 20;9:e11742. doi: 10.7717/peerj.11742 (PMC8300531; doi:10.7717/peerj.11742)
Supplement: Supplemental Information 8 [file peerj-09-11742-s008.pdf]

Summarising Tracking Data

Matt Crane, Inês Silva, Benjamin Michael Marshall, Colin Thomas Strine

24/08/2020

We tracked 7 [SPECIES NAME] individuals for an average of 649.7 ±112.27 days. During the tracking period, we located individuals on average every 8.51 ±0.09 hours (mean time lag), and detected 537 ±108.2 moves per individual with a mean step length of 60.98 ±1.63 m.

Table 1: Summary of animal tracking

Animal ID	# datapoints	Duration (days)	Mean time lag ±SE (hours)	# moves	Mean step length ±SE (m)
AF017	2245	774.97	8.29 ±0.11	747	31.84 ±1.96
AM006	2173	723.05	7.99 ±0.17	553	71.88 ±4.23
AM007	969	320.66	7.95 ±0.29	225	72.24 ±6.71
AM015	1944	680.13	8.4 ±0.18	595	71.05 ±4.74
AM018	3122	1176.10	9.04 ±0.28	1018	75.72 ±4.06
JM013	1497	561.19	9 ±0.17	390	48.35 ±3.31
JM019	890	311.79	8.42 ±0.21	231	43.22 ±4.97

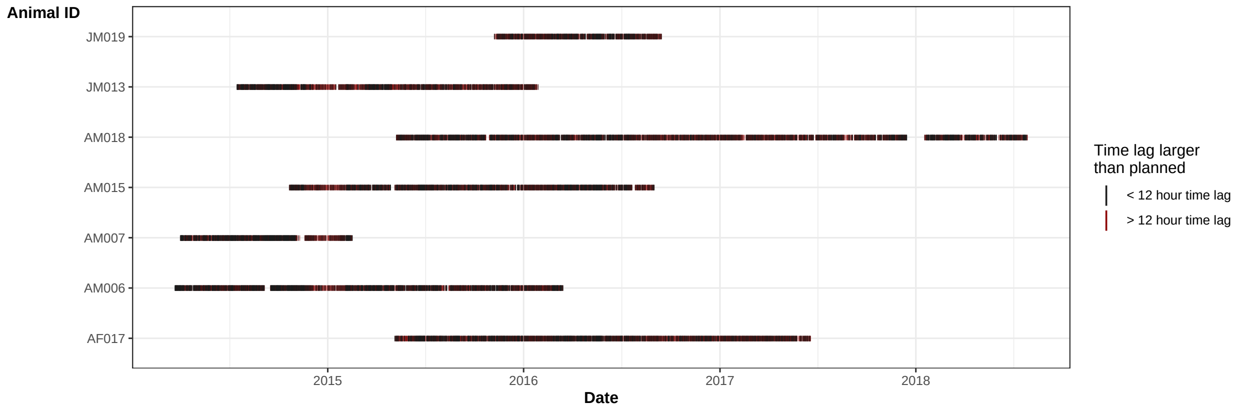

Figure 1: The tracking duration of all indivudals.

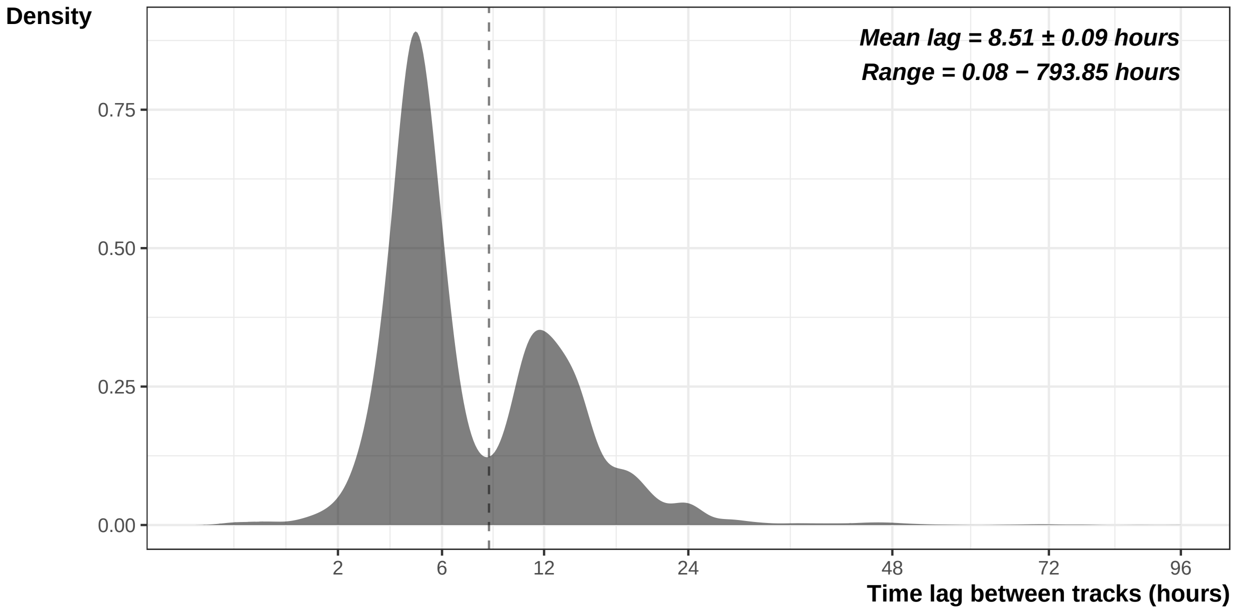

Figure 2: The distribution and mean (dashed line) lag time between tracks. Note, x-axis is square-rooted and truncated at 96 hours
